# The Role of Atypical Chemokine Receptor D6 (ACKR2) in Physiological and Pathological Conditions; Friend, Foe, or Both?

**DOI:** 10.3389/fimmu.2022.861931

**Published:** 2022-05-23

**Authors:** Arezoo Gowhari Shabgah, Farhad Jadidi-Niaragh, Hamed Mohammadi, Farnoosh Ebrahimzadeh, Maziar Oveisee, Abbas Jahanara, Jamshid Gholizadeh Navashenaq

**Affiliations:** ^1^School of Medicine, Bam University of Medical Sciences, Bam, Iran; ^2^Immunology Research Center, Tabriz University of Medical Sciences, Tabriz, Iran; ^3^Department of Immunology, Faculty of Medicine, Tabriz University of Medical Sciences, Tabriz, Iran; ^4^Department of Immunology, School of Medicine, Alborz University of Medical Sciences, Karaj, Iran; ^5^Non-Communicable Diseases Research Center, Alborz University of Medical Sciences, Karaj, Iran; ^6^Department of Internal Medicine, Faculty of Medicine, Mashhad University of Medical Sciences, Mashhad, Iran; ^7^Clinical Research Center, Pastor Educational Hospital, Bam University of Medical Sciences, Bam, Iran; ^8^Noncommunicable Diseases Research Center, Bam University of Medical Sciences, Bam, Iran

**Keywords:** ACKR2, cancer, inflammation, atypical chemokine receptor D6, infection

## Abstract

Chemokines exert crucial roles in inducing immune responses through ligation to their canonical receptors. Besides these receptors, there are other atypical chemokine receptors (ACKR1–4) that can bind to a wide range of chemokines and carry out various functions in the body. ACKR2, due to its ability to bind various CC chemokines, has attracted much attention during the past few years. ACKR2 has been shown to be expressed in different cells, including trophoblasts, myeloid cells, and especially lymphoid endothelial cells. In terms of molecular functions, ACKR2 scavenges various inflammatory chemokines and affects inflammatory microenvironments. In the period of pregnancy and fetal development, ACKR2 plays a pivotal role in maintaining the fetus from inflammatory reactions and inhibiting subsequent abortion. In adults, ACKR2 is thought to be a resolving agent in the body because it scavenges chemokines. This leads to the alleviation of inflammation in different situations, including cardiovascular diseases, autoimmune diseases, neurological disorders, and infections. In cancer, ACKR2 exerts conflicting roles, either tumor-promoting or tumor-suppressing. On the one hand, ACKR2 inhibits the recruitment of tumor-promoting cells and suppresses tumor-promoting inflammation to blockade inflammatory responses that are favorable for tumor growth. In contrast, scavenging chemokines in the tumor microenvironment might lead to disruption in NK cell recruitment to the tumor microenvironment. Other than its involvement in diseases, analyzing the expression of ACKR2 in body fluids and tissues can be used as a biomarker for diseases. In conclusion, this review study has tried to shed more light on the various effects of ACKR2 on different inflammatory conditions.

## 1 Introduction

Chemokines are small chemotactic cytokines produced by many cell types (e.g., immune cells, endothelial cells, and tumor cells) that are primarily involved in regulating the movement of immune and non-immune cells during normal immune surveillance, inflammatory situations, and development (1). These chemoattractant cytokines are divided into four subfamilies based on the location of conserved cysteine residues in their N-terminus: CXC, CX3C, CC, and C (1). Additionally, they may be categorized as homeostatic or inflammatory chemokines depending on the circumstances in which they are generated. In normal circumstances, homeostatic chemokines (e.g., CCL19, CCL20, and CCL21) are constitutively generated and control leukocyte movement. Inflammatory chemokines (e.g., CXCL8, CCL2, and CCL3) are generated in pathological situations and may function as secondary mediators when main pro-inflammatory substances such as IL-1β and TNF-α are present (2). Inflammatory chemokines play a significant role in the inflammatory response by recruiting immune cells to the site of damage. Besides their primary function in leukocyte recruitment, chemokines have been implicated in regulating several processes, including proliferation, angiogenesis, fibrosis, invasion, and metastasis (3, 4).

Chemokine receptors, which are considered members of a family of seven transmembrane G-protein-coupled receptors (GPRs), mediate the particular actions of chemokines on their target cells (1). These receptors have a highly conserved structure that includes a single polypeptide chain with three extracellular and intracellular loops, one external N-terminus domain essential for the specificity of ligand binding, and an intracellular C-terminus involved in receptor signaling with other motifs, such as the DRYLAIV motif between the second intracellular loop and the third transmembrane domain (1, 5, 6).

## 2 Atypical Chemokine Receptor 2

Apart from the conventional chemokine receptors, a subfamily of atypical chemokine receptors (ACKRs) has been discovered (7). These receptors are referred to as “atypical” because they have structural similarities with conventional chemokine receptors and have a high affinity for their ligands but lack the ability to stimulate cell migration. Indeed, these receptors lack the conventional DRYLAIV motif and, unlike the canonical chemokine receptors, do not initiate GPCR activation in response to chemokine interaction. Rather than that, ACKRs internalize and transport chemokines to the degradative compartment, thus regulating the concentration and bioavailability of chemokines. The ACKR family consists of four receptors: DARC (ACKR1), D6 (ACKR2), CXCR7 (ACKR3), and CCX-CKR (ACKR4) (8). These receptors act as decoys, scavengers, transporters, and depots for ligands, and they control how many of those ligands are available to their ligands (9).

### 2.1 ACKR2: Genomic Structure and Variations, Expression, Signaling, and Tissue Distribution

ACKR2, also known as D6 or chemokine binding protein 2 (CCBP2), was first cloned by Nibbs et al. in 1997. The human *ACKR2* cDNA showed 86% similarity and 71% identity to the murine *Ackr2* gene, and regarding protein, both differ from other chemokine receptors as they replace the highly conserved DRYLAIVHA motif with DKYLEIVHA in human ACKR2 and mouse Ackr2, bringing an extra charged residue into the region (10). According to the NCBI Gene database (https://www.ncbi.nlm.nih.gov/gene/1238), the *ACKR2* gene comprises three exons mapped to chromosome 3p22.1, a region of the genome that contains a cluster of genes encoding chemokine receptors. Moreover, according to the UniProt database (https://www.uniprot.org/uniprot/P21741), ACKR2 protein contains 384 amino acids, of which amino acid 19 is N-glycosylated, which is considered dispensable for ligand binding and high expression (11), and there is a disulfide bond between amino acid 117 and 195. Other than the conventional *ACKR2* gene, it was also defined as an SNP in this gene called rs2228467 (ACKR2-V41A allele), in which a single amino acid change at position 41 in its binding site, i.e., the first three extracellular loops, sets V41A apart from wild-type ACKR2. It has been shown that this allele is structurally similar to the wild-type ACKR2, and there was no noticeable variation in the predicted folding patterns of proteins, and both have similar expression levels in transfected CHO-k1 cells. When compared to wild type, ACKR2-V41A is estimated to have minor hydrophilicity. In terms of binding affinity, the binding affinity for CCL2 and CCL4 is decreased in V41A, leading to a decreased scavenging efficiency of this allele. Regarding receptor recycling, it was also demonstrated that ACKR2-V41A has decreased receptor recycling and expression levels on the cell surface for a long period of time. Furthermore, Ca^2+^-dependent signaling is considerably lower in CCL2-activated cells that express the ACKR2-V41A allele than in the wild type, resulting in decreased signaling following CCL2 activation in this allele. These variations in binding, recycling, and signaling have shown different efficiency in various conditions, possibly leading to different responses in diseases (12). When it comes to disease and inflammatory conditions, rs2228467 has been linked to altered chemokine levels in the bloodstream and cerebrospinal fluid (CSF). According to research conducted in 2017 with 8,293 Finnish individuals, elevated levels of the chemokine CCL11 (a powerful eosinophil attractant) were associated with the rs2228467 SNP and an increased risk of Crohn’s disease, ulcerative colitis, and multiple sclerosis (13). In separate research, rs2228467 was also shown to be related to an increased risk of coronary heart disease and an elevated monocyte count in the bloodstream of 11,014 participants from the electronic Medical Records and Genomics Network (eMERGE) (14). In addition, rs2228467 was proposed as a possible determinant of a risk factor for developmental disorders after an examination of over 700 mother–infant pairs, maternal mid-gestational serum, and neonatal bloodspot-derived immune mediators (15). Another study published in 2017 that examined data from the National Human Genome Research Institute (NHGRI) discovered that rs2228467 is associated with an increased risk of Alzheimer’s disease (AD) (16). A possible underlying mechanism for the involvement of rs2228467 in AD is that this variant has weaker CCL2 scavenging efficiency compared to the wild type, resulting in persistent inflammation in the brain (12). rs4683336 is another SNP found in the *ACKR2* gene, which is located in the first intron of the gene, where it harbors regulatory elements that can modulate gene transcription. When it comes to diseases, at both the genotype and haplotype levels, it has been proven that the SNP rs4683336 is substantially linked to the histological grade of inflammation in HCV-induced liver disease. Furthermore, the relationship between rs4683336 and hepatic inflammation seems to be strong, as shown by the fact that it has been established in both qualitative and quantitative genetic analyses. However, the scavenging effect of rs4683336 on CC chemokines needs to be clarified (17).

Placental trophoblasts (syncytiotrophoblasts), some lymphocytes (such as innate B cells, i.e., splenic marginal zone B cells and B1 cells), myeloid progenitor-derived cells (such as mast cells and dendritic cells), blood endothelial cells, and lymphatic endothelial cells (LECs)—the major ACKR2-expressing cells in adults—have been shown to express ACKR2 (18–22). According to Nibbs et al.’s study, human LECs but not vascular endothelial cells express the ACKR2 gene in non-inflamed tissues, suggesting a functional heterogeneity in the lymphatic vasculature since the receptor was found on a fraction of lymphatics (18). ACKR2 was once thought to be a non-signaling chemokine receptor because of its “atypical” properties. As it turned out, ACKR2 is capable of internalization, as well as the ability to remove ligands from the cytoplasm and return them to the cell membrane *via* activating a β-arrestin-dependent pathway (23, 24). It has been reported that ACKR2 ligation induces a β-arrestin1-dependent, but independent of the G-protein signaling pathway, resulting in the phosphorylation of the actin-binding protein cofilin through the Rac1/p21/PAK1/LIMK1 cascade (24, 25). For the increased quantity of ACKR2 protein at the cell surface, as well as for the chemokine-scavenging function of this receptor, this signaling pathway needs to be activated. Therefore, it is concluded that ACKR2 is a signaling receptor that regulates chemokine-mediated responses in inflammation and immunity through a specific signaling pathway that is different from that of other chemokine receptors (24).

This receptor is a highly versatile ACKR capable of binding to the majority of inflammatory CC chemokines, including CCL2, CCL3, CCL4, CCL5, CCL7, CCL8, CCL11, CCL13, CCL14, CCL17, and CCL22 (10, 26). Although Lee et al. have mentioned that ACKR2 does not bind to CXC, C, CX3C, and other hemostatic CC chemokines, it has been shown that ACKR2 likely interacts with CXCL10 and CXCL14 (27, 28). It has been shown that in ACKR2-expressing cells, the vast majority (more than 80%) of ACKR2 protein is located in internal endosomes, and 10%–20% of the ACKR2 protein is on the cell surface (11). Antibody-feeding studies have established ligand-independent internalization (like CCR5) and recycling of ACKR2 (unlike CCR5), allowing ACKR2 for high-affinity binding, internalization, and degradation of ligands (29). Chemokines seem to elicit the expression of ACKR2 on the cell surface, allowing cells to better scavenge and internalize ligands. It is suggested that ACKR2 is a constitutively active scavenging receptor for inflammation-induced CC chemokines (23, 30). Since it has been confirmed that ACKR2 is not transcriptionally regulated in a variety of cellular settings, optimizing its scavenger function in response to ligands suggests a quick and unique strategy by which ACKR2 may control inflammation (23). Mechanistically, it has been shown that ACKR2 regulation involves a high-energy process driven by Rab11 vesicles, in contrast to canonical chemokine receptors, which are downregulated by homologous ligands (23).

Other than mechanical up- or downregulation of ACKR2, it has been shown that this receptor is also regulated by transcription factors and miRNA like other genes. In this regard, it has been confirmed that ACKR2 expression can be regulated by GATA1, which is a prototypic hemopoietic transcription factor. The identification of this transcription factor justifies the expression of ACKR2 in myeloid-derived cells, including megakaryocytes, myeloid progenitors, mast cells, and DCs. In this respect, it was shown that transfection of myeloid cells with the *GATA1* gene induced ACKR2 expression in these cells, suggesting that the *ACKR2* promoter possibly contains a binding site for GATA1 (22). Besides GATA1, microRNAs also regulate ACKR2 expression. For example, miR-146b and miR-10b are considered regulators of this gene (31, 32).

## 3 Physiological Function of ACKR2

**Figure 1:**
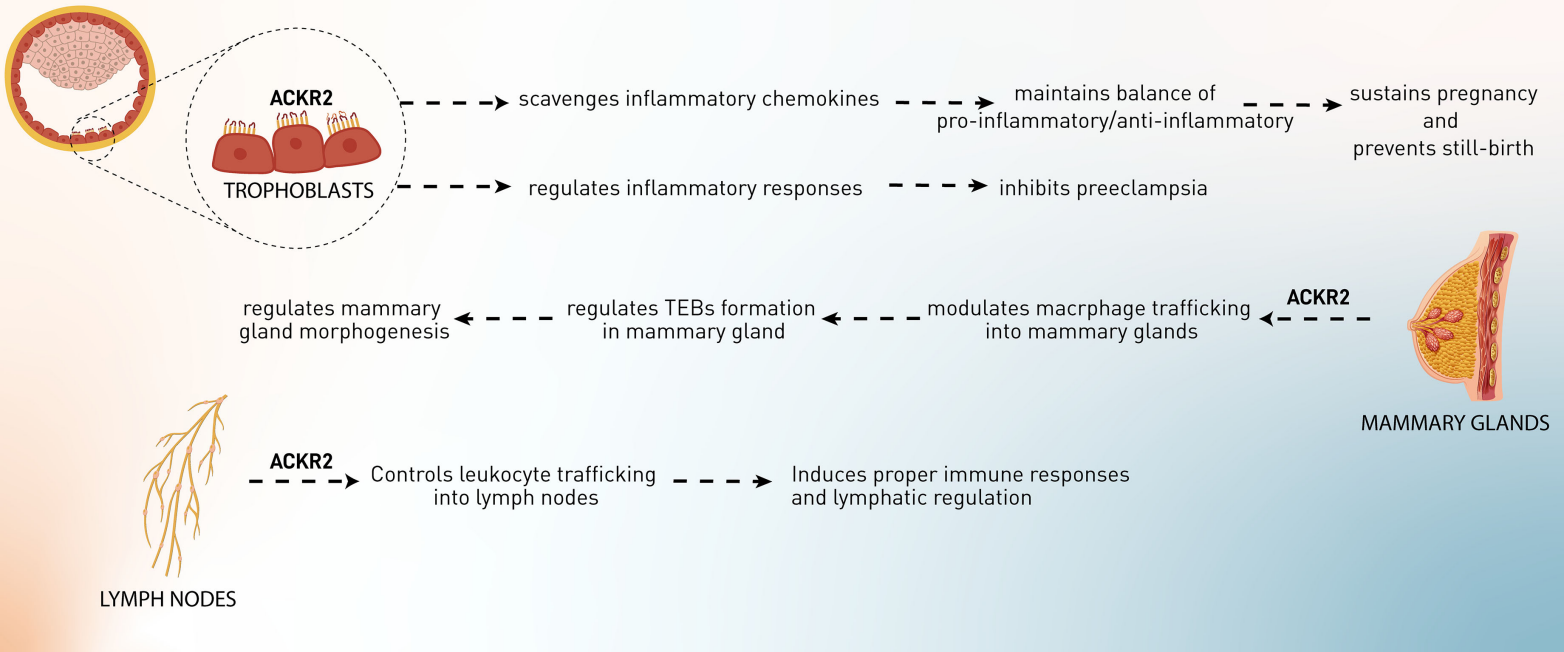
The role of ACKR2 in the physiological processes, including pregnancy, development of mammary glands, and lymphatic regulation.

As has been mentioned, ACKR2 acts as a scavenging receptor for chemokines. Therefore, it can be involved in physiological activities in the body (**Figure 1**). Since leukocyte recruitment is mediated by chemokines, these mediators contribute to several mechanisms in the body. Therefore, the regulation of recruitment and infiltration of leukocytes might be important in hemostatic functions.

### 3.1 The Role of ACKR2 in the Pregnancy

Implantation is a process in which maternal inflammatory responses are developed and sustained during pregnancy. Regulation of the interaction between maternal leukocytes and fetal trophoblasts is vital for successful implantation, placental development, and pregnancy maintenance. Therefore, abnormal placentation may result from a breakdown of this control (33). Studies in both mice and humans have shown that abnormal placentation is a significant cause of maternal and perinatal mortality and morbidity (34). Placental recruitment of leukocytes occurs by attracting certain leukocytes from the circulation. The chemokine family is responsible for a considerable proportion of leukocyte recruitment. In the human placenta, maternal and fetal cells express a variety of chemokines at particular anatomical regions, and placental leukocytes express chemokine receptors and react to chemokines. Functional chemokine receptors, such as CCR1 and CCR2, have also been found in cytotrophoblasts. It has been hypothesized that chemokines attract immune cells into the placenta and support the invasion and placement of trophoblasts (35, 36). Placental leukocytes express chemokine receptors, including CCR1 and CCR2, on the surface of placental leukocytes, which may guide the migration of these cells in response to inflammatory chemokines generated in the placenta (37–39). As has been discussed, conventional chemokine receptors can trigger inflammatory cascades by binding to CC chemokines, whereas ACKR2 does not (40). Since trophoblasts are the richest source of ACKR2, it has been postulated that this receptor might play an indispensable role in these cells (41). Martinez et al. have suggested the significance of ACKR2 receptors in regulating the pro-inflammatory/anti-inflammatory balance at the maternal–fetal interface (42). ACKR2 is assumed to be inevitable in mice for the normal conception of undisturbed females, and it might inhibit inflammation-induced abortion and help the survival of implanted allogeneic embryos (42, 43). According to the evidence, ACKR2-knockout pregnant mice exposed to LPS or anti-phospholipid antibodies have increased pro-inflammatory CC chemokines, elevated placental leukocyte infiltration, and subsequently increased fetal loss (41–43). It was also shown that *Ackr2* loss in unchallenged DBA/1j mice increases the rate of stillbirth and neonatal mortality, causes structural problems in the placenta, and reduces fetal weight in pregnant mice. Placental abnormalities are caused by the loss of Ackr2 in fetal cells. As a result, the trophoblast cells of the placenta need ACKR2 to scavenge chemokines, and *ACKR2* deficiency may lead to aberrant placental morphology and decreased newborn survival (41). Other than the murine model, human trophoblast cells also highly express ACKR2, at least tenfold more than other chemokine receptors, including CCR1, 2, 3, and 5, but not CCR4, suggesting that the *ACKR2* gene is more actively expressed than genes encoding conventional receptors for inflammatory CC chemokines or even other ACKRs. ACKR2 efficiently internalizes CCL2 into the cells, showing that this ACKR plays a pivotal role in inhibiting inappropriate inflammatory responses to maintain pregnancy and suppress preterm delivery or pregnancy loss (41).

Besides maintaining pregnancy, ACKR2’s effects on pregnancy complications have also been observed. In preeclampsia, systemic inflammatory responses are mediated by chemokines (CCL2, CCL7, and CCL11), cytokines (IL-6), leukocytes, the coagulation cascade, and the complement system. Regarding ACKR2, although it has been shown that placental ACKR2 is decreased at transcriptional and protein levels in preeclampsia in Cho et al.’s study, this downregulation has not been observed in Tersigni et al.’s investigation. Cho et al. have hypothesized that ACKR2 downregulation leads to higher inflammatory responses, resulting in excessive recruitment of macrophages to the maternal–fetal interface and an inappropriate response to systemic inflammation (44). In contrast, Tersigni et al. postulated that in preeclampsia, increased levels of pro-inflammatory chemokines in the blood and placenta might bind to the syncytiotrophoblast membrane’s ACKR2 decoy receptor, resulting in an increased concentration of the scavenger on the syncytiotrophoblast cell membranes as a result of its mobilization from the intracellular pool. Simultaneously, cytoskeleton damage in trophoblast cells, most likely as a result of syncytiotrophoblast oxidative stress, impairs ACKR2 function, which is already stressed owing to the elevated chemokine levels. This explanation is consistent with the *in vitro* discovery of increased ACKR2 expression but decreased CCL2 scavenging in cytotrophoblast membranes separated from preeclampsia placentae. In conclusion, syncytiotrophoblast stress and cytoskeleton impairment may occur at various times during both early and late preeclampsia, leading to impairment of the cell cytoskeleton, ACKR2-mediated chemokine degradation, and ACKR2 receptor recycling, resulting in inadequate regulation of the inflammatory environment at the maternal–fetal interface (45). Additional research is required to elucidate the role of ACKR2 scavenging in the etiology of preeclampsia and to identify novel treatment approaches targeting the ACKR2 molecular system. Indeed, it is plausible to infer that optimizing ACKR2 function may help mitigate the excessive inflammatory response seen in preeclampsia. Besides the role of ACKR2 in preeclampsia, it was proposed that levels of ACKR2 expression in the different types of preeclampsia (early-onset preeclampsia, late-onset preeclampsia, and preterm birth) might be a biomarker of the condition’s progress. ACKR2 mRNA and protein levels in the early-onset preeclampsia and the preterm birth group but not late-onset preeclampsia were significantly reduced compared with normal subjects. Recently, evidence has suggested that ischemia and hypoxia in placental tissue are key features of preeclampsia. These conditions might induce some factors from the placenta, which lead to downregulation of ACKR2 and higher expression of CCL2 in trophoblasts, requiring further investigation. Collectively, hypoxia in the placenta induces downregulation of ACKR2 and CCL2-related inflammation, leading to preeclampsia and probably fetal loss (43).

### 3.2 The Role of ACKR2 in the Mammary Gland Development

The development of mammary glands is mediated through a process called branching morphogenesis, which leads to ductal epithelial networks. In this process, proliferative structures, known as terminal end buds (TEBs), are developed at the epithelial ducts and promote network formation. A stromal component composed of fibroblasts, extracellular matrix (ECM), adipocytes, and immune cells leads to the achievement of this process. Mammary glands and surrounding TEBs contain macrophages, and their phagocytic and cytokine-producing characteristics are essential for several tissue remodeling events during developmental processes. In macrophage-deficient animals, mammary gland development is substantially disrupted, accompanied by abnormal TEB formation, ductal elongation throughout puberty, and lobuloalveolar development during pregnancy. In summary, these investigations demonstrate that macrophages play a critical role in the control of ductal branching in the developing mammary gland (46, 47). Macrophages are located mostly in the neck region or inside the TEBs, where they release proteinases and growth factors that aid in the promotion of collagen fibrillogenesis (collagen formation). According to the observations, different stages of the development of the mammary gland in virgin mice are markedly characterized by the expression of CC chemokines, including CCL2, CCL4–CCL9, CCL11, CCL17, CCL22, and CCL26) and their receptors (CCR1, CCR2, and CCR5). These chemokines and chemokine receptors recruit and infiltrate macrophages and other leukocytes into the TEBs, leading to network formation. It has been shown that Ackr2 is expressed in the developing mammary glands by stromal cells such as fibroblasts. Ackr2 expression in this microenvironment regulates CC chemokine (CCL7/CCR1)-dependent trafficking of CD206^+^ macrophages into the mammary glands and subsequently controls the process of mammary gland branching morphogenesis during development. Ackr2-deficient mice have displayed premature development of mammary glands. This was linked to elevated infiltration of macrophages into the developing gland, resulting in an increased density of the ductal epithelial network. These findings show that ACKR2 is a key regulator of branching morphogenesis in a variety of biological situations and support the involvement of chemokines, playing critical roles in postnatal development processes (48, 49). Furthermore, bioinformatic investigation of the premature puberty (CTD Gene-Disease Associations) dataset using Harmonizome indicated that CCL7 and ACKR2 are both related to precocious puberty in children, which adds more weight to this conclusion (50).

### 3.3 Lymphatic Regulation

The lymphatic vessels (lymphatics) and LECs play a vital role in the flow of fluid and leukocytes such as APCs from the peripheral tissues to the secondary lymphoid organs, especially lymph nodes. Human LECs but not vascular endothelial cells have been shown to express ACKR2 within non-inflamed tissues. Since this receptor has been found in a subset of lymphatic vessels, proposing that the heterogeneity in the expression of ACKR2 within the lymphatic vasculature might be a sign of function in this microenvironment. For example, only afferent lymphoid vessels in regions near the epidermis and deeper in the dermis express ACKR2 in the human skin sections. ACKR2 is also expressed in small and large intestines (in the villi), colon (lamina propria mucosa and muscular layer), appendix (in lamina muscularis externa), and other secondary lymphoid organs, including spleen (in red pulp) and tonsils (on sinuses like channels and parafollicular areas) (51). According to McKimmie et al.’s study, ACKR2 expression in human dermal LECs was upregulated in response to VEGF-D, TGF-β, IL-6, type I interferons, and IFN-γ. In contrast, IL-1α downregulated the expression of ACKR2 (52). It was shown that ACKR2, by scavenging inflammatory CC chemokines, restricts the interaction, access, and inappropriate infiltration of inflammatory cells in the lymph nodes, thus contributing to efficient antigen presentation by DCs and selective presentation of CCR7 ligands, i.e., CCL19 and CCL21 to mature DCs (52). Moreover, ACKR2 competes with CCR2 in binding CCL2, regulating macrophage proximity to lymphatic vessels and contributing to controlling lymphangiogenesis and proper immune response (53). Therefore, ACKR2 forms functional gradients that promote leukocyte recruitment into inflamed tissues, control their trafficking into lymph nodes, and regulate lymphatic vessel density, which avoids inappropriate perilymphatic infiltration of inflammatory leukocytes at draining lymph nodes and peripheral inflamed sites and consequently inhibits lymphatic congestion (54).

### 3.4 Resolution of Inflammation

When the body’s tissues are exposed to harmful stimuli such as infections, damaged cells, or irritants, inflammation occurs. It is a defensive reaction involving immune cells, blood vessels, and molecular mediators, and it is a complicated biological response involving several factors. An inflammatory response’s primary goal is to remove the source of cell damage, which helps remove necrotic cells and tissues that have been destroyed by the initial harmful stimuli as well as the inflammatory response, leading to tissue healing (55). During this process, chemokines play pivotal roles due to their ability to recruit inflammatory cells, including neutrophils, monocytes, lymphocytes, and other cells such as fibroblasts. These cells mediate inflammatory responses, leading to the removal of harmful stimuli. The process of quenching inflammatory responses after the removal of stimuli is called the resolution of inflammation, and it is considered a physiological and homeostatic procedure (55). A broad spectrum of inflammatory chemokines, including CCL2–8, CCL11–13, CCL17, and CCL22, are scavenged by ACKR2, which aids in the resolution phase of inflammation and restricts the escalation of immune responses. Defects in the process of inflammation resolution mechanisms such as ACKR2-dependent scavenging can be deleterious due to the development of chronic inflammation (56). In the upcoming section, the diseases that result from defects in ACKR2-related chemokine scavenging are discussed.

## 4 The Role of ACKR2 in Diseases

Numerous investigations employing human samples or ACKR2-deficient animals have shown that ACKR2 expressed by lymphatic vessels plays a critical role in inflammatory diseases and malignancy. While the increasing consensus is that ACKR2 works as a negative regulator of inflammation, conflicting findings have been reported about its involvement in the modulation of adaptive immune responses and the development of autoimmune disease.

### 4.1 ACKR2 and Cancer

#### 4.1.1 Tumor-Suppressive Functions of ACKR2

The tumor microenvironment is the main interaction site between cancer cells and the host immune system. Through interactions between chemokines and chemokine receptors, diverse immune cell subsets are attracted to the tumor microenvironment, and these populations have unique impacts on tumor growth and treatment outcomes (57).

**Figure 2:**
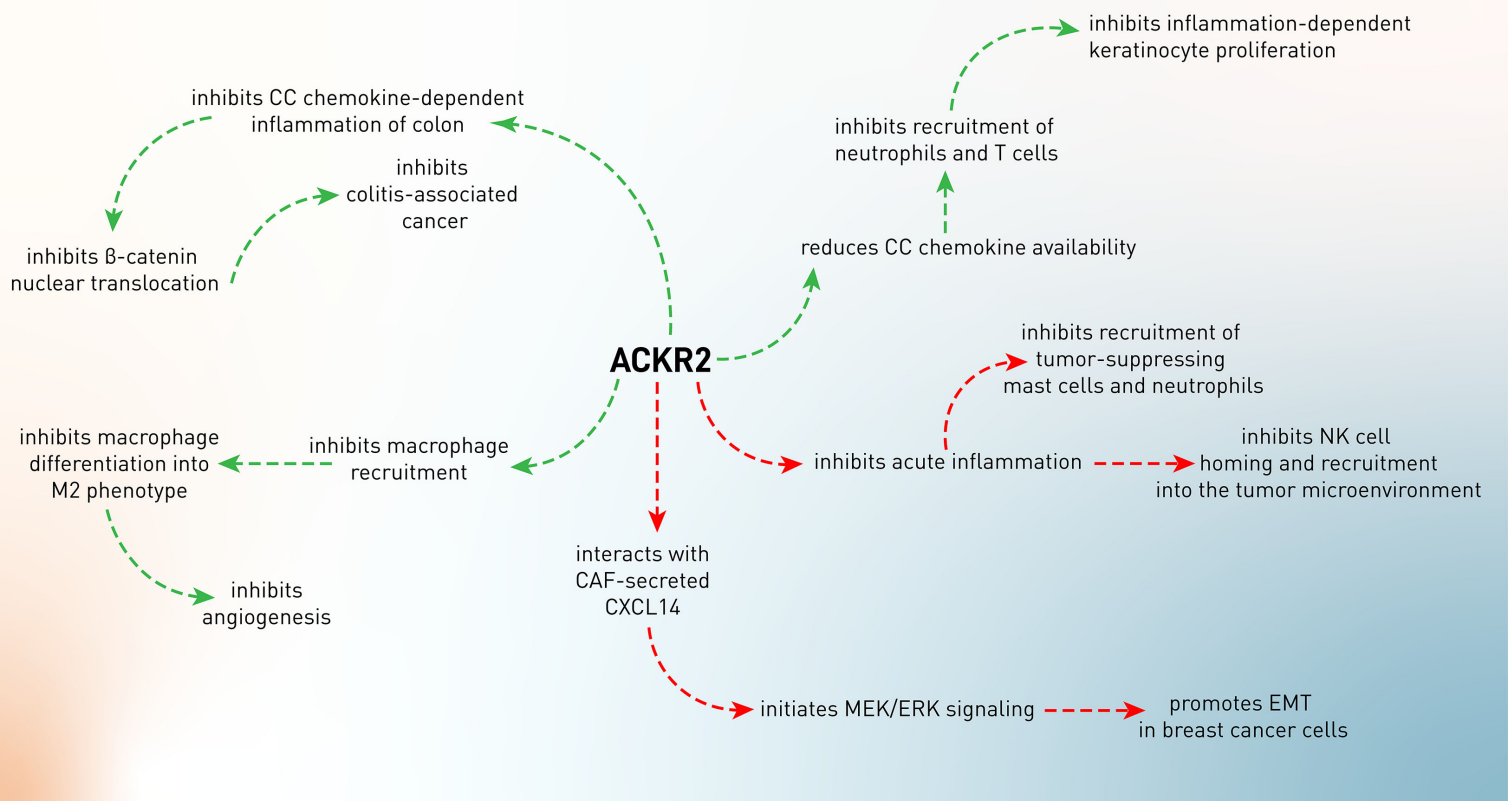
The conflicting role of ACKR2 in tumor development. The green arrows indicate the tumor-suppressing role of ACKR2, while its tumor-supportive roles were shown by the red arrows.

A group of chemokines, including CCL2, CCL3, and CCL5, working through the CCR1 to CCR5 receptors, plays a critical role in determining inflammatory responses that may be tumorigenic. These proinflammatory CC chemokines have been associated with several facets of cancer biology and possibly play a key role in *de novo* tumor development. CCL3 is highly elevated in tetradecanoyl phorbol acetate/dimethylbenz(a) anthracene (TPA, a tumor-promoting agent for cutaneous malignancy/DMBA)-induced inflammation in B6/129 ACKR2-deficient mice, leading to recruitment and infiltration of polymorphonuclear cells (PMNs) and CD3^+^ T cells into papilloma. This hyperactive inflammatory response enhanced papilloma formation and made ACKR2-deficient mice more susceptible to the higher proliferation of keratinocytes (**Figure 2**). In contrast, transgenic expression of ACKR2 in keratinocytes reduced inflammation and decreased the susceptibility of mice to skin tumors. These results indicated that ACKR2, by inhibiting chronic inflammation, suppressed the formation of skin tumors, which possibly results from its scavenging capability of CCL3 (58).

The two most common types of inflammatory bowel illness are Crohn’s disease and ulcerative colitis (IBD). The production of pro-inflammatory chemokines is common in people with IBD. Additionally, the persistent mucosal inflammation seen in IBD has been linked to a higher incidence of colon cancer, also called colitis-associated cancer. Inflammatory cytokines have also been shown to play a role in the development of colonic inflammation into colon cancer. If pro-inflammatory chemokines are not properly controlled, it may lead to persistent intestinal inflammation and even cancer (59). In an experiment, it has been shown that ACKR2^–/–^ mice develop experimental colitis by increased production of inflammatory chemokines (CCL3, CCL5, CXCL1, CCL2, and CXCL2) and elevated leukocyte infiltration (T cells, macrophages, B cells, and DCs) in the inflamed mucosa of the colon (60). These high levels of inflammation promoted the progression and incidence of colitis-associated cancer, which was characterized by higher levels of β-catenin nuclear translocation and higher epithelial proliferative activity (60). These data indicate the anti-inflammatory effects of ACKR2, leading to abrogation of inflammation-associated tumor development (60).

As discussed in the former paragraph, ACKR2 is considered a regulator of the chemokine system, specifically boosting the clearance of CC chemokines and modulating tumor-associated inflammatory responses. It has been shown that 4-nitroquinoline-1-oxide (4NQO, a chemical carcinogen)-treated ACKR^–/–^ mice develop high levels of IL-1β, IL-6, IL-12, IL-17, TNF-α, CCL2, CCL3, CCL4, CCL5, CCL12, CCR1, CCR2, CCR5, FGF, and VEGF in oral SCC lesions, leading to higher infiltration of Ly6G^+^ neutrophils, mast cells, and F4/80^+^ macrophages in tumor tissues and proposing an unleashed inflammation in the tumor site. This inflammatory milieu results in the creation of highly proliferative tumor cells (61). The primary role of macrophages is to protect the body against external infectious pathogens. However, when macrophages are recruited into neoplastic tissues, they differentiate into tumor-associated macrophages (TAMs) and mainly M2 macrophages, which exhibit a distinct polarization, favoring tumor development and angiogenesis over anti-tumor immune responses, which are primarily mediated by M1 macrophages. CCL2 and CCL5 are responsible for the recruitment of macrophages into the tumor microenvironment and tumor development (62). Therefore, macrophage recruitment and polarization into the M2 subtype lead to tumor invasiveness. In Kaposi sarcoma, it has been shown that activation of the K-Ras-B-Raf-ERK pathway results in downregulation of ACKR2 expression, unleashing CCR2-mediated recruitment and activation of M2-like phenotype of macrophages, which support angiogenesis and tumor growth *via* producing VEGFA (63).

#### 4.1.2 The Tumor-Supportive Activity of ACKR2

In addition to the tumor-inhibitory effects of ACKR2, it has also been shown that this decoy chemokine receptor has been involved in tumor progression (**Figure 2**). As a matter of fact, chemokines play a pivotal role in the recruitment of potent anti-tumor immune cells, including T cells and NK cells. In the meantime, CCL2 is one of the chemokines involved in immune cell recruitment, especially of NK cells (64). Therefore, CCL2 expression in the tumor microenvironment attracts CCR2-expressing KLRG^+^ NK cells, leading to enhanced tumor-homing efficiency of NK cells. ACKR2^–/–^ mice have been demonstrated to have increased killing activity of NK cells through enhanced CCR2 expression and NK infiltration into the CCL2-expressing melanoma tumor microenvironment. As a result, ACKR2 expression in the tumor microenvironment decoys CCL2 and impairs the infiltration of NK cells (65).

Despite having the tumor-promoting functionality of chronic inflammation, acute inflammation can exert an essential role in tumor surveillance (66, 67). Sometimes, the anti-tumor function of acute inflammation has been referred to as mast cells (68, 69). These cells play conflicting roles in tumor development. Several investigations in rodents and humans have shown a link between the presence of mast cells in the tumor microenvironment and prognosis, emphasizing the complexities of mast cell-mediated effects (69). By increasing inflammation and angiogenesis, tumor-associated mast cells play a role in the development of many malignancies (69). On the other hand, mast cells’ presence is associated with a better prognosis in human colon cancer (70). In ACKR2-deficient mice with colitis-associated colon cancer, it has been shown that there is an increased infiltration of mast cells into the tumor microenvironment (71). This resulted from sustained expression of CCR2 and CCR5 in the absence of ACKR2 due to the abrogation of ACKR2’s chemokine scavenging function and resolution of inflammation. Therefore, elevated CCR2 and CCR5 led to the infiltration of mast cells and production of leukotriene B4 (LTB4), which consequently recruited CD8^+^ T cells into the tumor microenvironment. Moreover, mast cells present tumor antigens to T cells and promote IFN-γ production, resulting in the inhibition of tumor growth and mediating immune surveillance (71).

Besides mast cells, neutrophils also exert anti-tumor roles in cancer development beyond their inflammatory functions (72). In another study using ACKR2-deficient mice, it has been shown that inactivation of ACKR2 leads to higher levels of inflammatory chemokine receptors and enhances the release of neutrophils with more incredible anti-metastatic action from the bone marrow (73). ACKR2 loss is linked to enhanced initial tumor development and protection against metastasis in a NeuT-driven primary mammary carcinogenesis. In mice orthotopically implanted with 4T1 mammary cancer and intravenously injected with B16F10 melanoma cell lines, ACKR2 loss resulted in neutrophil-mediated protection against metastasis. Thus, ACKR2 is a critical checkpoint of myeloid development and function in mice, and its targeting activates neutrophil anti-metastatic activity. Taken together, these findings suggest that genetic inactivation of ACKR2 in early hematopoietic precursors leads to a faster maturation rate of neutrophils, which are more effectively mobilized and attracted to metastatic lesions, possibly *via* CCR1, CCR2, and CCR5, where they exert increased anti-metastatic action (73).

Tumor cells undergo an epithelial-to-mesenchymal transition (EMT) during the early stages of the metastasis process, losing cell-to-cell connections and epithelial properties in favor of mesenchymal ones that enable them to invade and metastasize to the surrounding or distant tissues. In this process, several transcription factors, including Twist, Slug, Snail, Gsc, and Zeb, are involved, leading to downregulation of epithelial markers (E-cadherin and cytokeratin) and upregulation of mesenchymal markers (vimentin, α-SMA, matrix metalloproteinases) (74). Cancer-associated fibroblasts (CAFs) are the important cells in the tumor microenvironment that mediate EMT by producing mediators such as CXCL14 (75). It has been shown that CAF-secreted CXCL14 suppresses epithelial markers and induces mesenchymal phenotypes in MCF-7 breast cancer cell-bearing mice, leading to the colonization of MCF-7 cells in the lungs of mice. Moreover, CAF-secreted CXCL14 showed similar effects on other breast tumor cells, i.e., SKBR3 and 4T1 cells, in a paracrine manner. Mechanistically, it has been confirmed that direct interaction of CXCL14 with ACKR2 initiates MAPK/ERK signaling and promotes EMT in SKBR3 cells, which is conversely inhibited upon downregulation of ACKR2, suggesting that CXCL14 mediates EMT and metastasis in breast cancer cells that depend on ACKR2 (27). However, the exact interaction or interplay of CXCL14 with ACKR2 or other chemokine receptors needs to be clarified.

#### 4.1.3 ACKR2, as a Biomarker in Cancer

As discussed, LECs are the main expressors of ACKR2 in the adult human body. Therefore, analyzing vascular tumors in terms of ACKR2 expression to find the origin of the tumor might be promising in detecting the type of cancer. In this regard, Nibbs et al. showed that ACKR2-immunoreactive tumor epithelial cells might be exclusively derived from LECs, suggesting that ACKR2 can be useful in detecting lymphatic tumor cells (51).

Cervical cancer is the female reproductive system’s second most prevalent malignancy. Regional lymph node metastases are a critical prognostic factor in individuals with cervical cancer. Around half of the cervical cancer patients with pelvic lymph node metastases will have a disease recurrence, with the majority suffering from uncontrolled tumor growth. The reported 5-year survival rate for individuals with a recurrence is between 3% and 13%, and prognostic indicators for lymph node metastases in patients with cervical cancer are lacking (76). Analyzing the association of ACKR2 with the prognosis of cervical squamous cell carcinoma (CSCC) showed that ACKR2 expression is reduced in CSCC compared to the normal cervix, and its expression was negatively related to tumor size. Moreover, patients lacking ACKR2 expression were found to experience shorter recurrence-free and overall survival times (77). Moreover, examination of other ACKRs in CSCC showed that expression of CCX-CKR and DARC was also negatively correlated with lymph node metastasis. Therefore, these ACKRs have been proposed as independent prognostic factors for predicting CSCC progression (77).

Although there is no defined correlation of ACKR2 expression with other carcinogenic factors in cervical cancer, ACKR2 downregulation has been accompanied by dysregulation of metastatic factors such as MMP9 and VEGF in breast cancer. In this cancer, expression of ACKR2 was accompanied by ACKR1 and ACKR4 which were negatively correlated to axillary lymph node metastasis of breast tumor cells, suggesting favorable outcomes for co-expression of ACKR2, DARC, and CCX-CKR in breast cancer (78, 79). Furthermore, patients lacking these chemokine receptors showed higher expression of VEGF and MMP9, indicating the anti-tumor functions of ACKRs in breast cancer (78). The anti-tumor effects of ACKR2 on breast cancer have been attributed to its anti-inflammatory function. ACKR2-induced breast tumor inhibition results mainly from decoying inflammatory chemokines (e.g., CCL2), inhibiting vascular density, and suppressing tumor-associated macrophage infiltration (80).

Since ACKR2 downregulation has been shown to contribute to the progression of colon adenocarcinoma, the correlation of ACKR2 expression in tumor tissues was analyzed to introduce its capability of being a biomarker in colon adenocarcinoma. Results showed that ACKR2 mRNA expression in colon adenocarcinoma tissues was decreased compared to unaffected mucosa. ACKR2 was also highly expressed in endothelial cells of the blood and lymphatic vessels of unaffected tissues compared with tumor tissues. Moreover, ACKR2 expression in tumor tissues was negatively correlated to tumor invasiveness, as its expression was lower in T3 and T4 grades than in T1 and T2 ones. All of the correlation was sex- and age-independent. In addition, ACKR2 expression was correlated to the expression of its counterpart ligand, i.e., CCL2, but not CCL5 and CCL8, suggesting that ACKR2 downregulation increases CCL2 in the tumor microenvironment to recruit more inflammatory cells and promote inflammation. CCL22, a Treg-recruiting chemokine, was another chemokine that was increased in the ACKR2-decreased tumor tissues, showing that ACKR2 downregulation also promotes tumor development by creating an immunosuppressive tumor microenvironment (81). Therefore, colon adenocarcinoma cells by downregulation of ACKR2 promote chronic inflammation and an immunosuppressive microenvironment to facilitate evasion of tumor cells from potent immune responses, suggesting ACKR2 as a potential biomarker for analyzing the development of colon adenocarcinoma.

In another study, ACKR2 expression was tracked to analyze the trend of expression through the conversion of an adenoma into an adenocarcinoma sequence. Results have demonstrated that the expression ratio of ACKR2 was decreased through normal to tumor and increasing depth of tumor invasion. Moreover, the expression ratio of ACKR2 was decreased along with elevating tumor grade (adenoma to adenocarcinoma), whereas its expression was elevated through normal to pathological in adenoma tissues. Collectively, during the adenoma procedure, ACKR2 expression was insignificantly increased, and then its expression declined insignificantly in the process of malignancy. Moreover, the highest downregulation of ACKR2 was at the point that adenoma cells are capable of being malignant (82). Thus, the divergence across studies is more likely due to changes in the molecular pathways leading to neoplastic transformation in colitis-associated cancer and sporadic colorectal cancer than to differences in receptor expression at the protein and mRNA levels.

Today, it is well known that chemokines, their receptors, and other mediators are part of an interactive network. Therefore, analysis of a set or group of mediators would be helpful in predicting disease progression and prognosis. In a study to analyze the profile of expression of chemokine ligands and receptors in salivary adenoid cystic carcinoma (SACC), it has been shown that the expression of ACKR2 alongside CXR1, CXCR2, CMKLR1, and ACKR4 was higher in patients without recurrence, while inflammatory chemokines such as CXCL11, CCL5, and CCL20 were higher in metastatic cell lines compared with non-recurrent and less-invasive cells (83). Although it has not defined the underlying mechanism of decoy chemokine receptors in this tumor type, this study indicates the importance of examination of a set of interactive chemokines to achieve a more accurate panel of biomarkers to evaluate tumor progression and invasion.

As has been mentioned, SNPs are recently considered predictors for diseases, particularly cancer. Regarding the impact of SNPs in the *ACKR2* gene on cancer, it has been shown that rs2228468 is correlated with breast cancer metastasis. In SNP rs2228468, serine substitutes with tyrosine at location 373, and it has been found that this SNP shows limited differential effects on chemokine degradation ability. Analysis of this SNP in breast cancer has concluded that SNP rs2228468 has been associated with TNM and that rs2228468 serves as an indication of a changed metastatic propensity in Han Chinese patients (84).

#### 4.1.4 The Applicability of ACKR2 in Therapeutic Approaches

It has already been stated that the B-Raf/MEK/ERK pathway mediates the downregulation of ACKR2, which leads to an increase in chemokine-mediated infiltration of macrophages and local activation of them toward a tumor-promoting M2-like phenotype (63), suggesting the importance of this pathway in the development of Kaposi sarcoma. Therefore, inhibition of this signaling pathway might provide a target for future treatment approaches. In this regard, it has been shown that administration of B-Raf inhibitor (PLX4032) and the MEK inhibitor (U0126) gives rise to ACKR2 upregulation and, subsequently, a decreased infiltration of TAMs in this malignancy, representing alternative or complementary therapeutic strategies (63).

### 4.2 Cardiovascular Diseases

**Figure 3:**
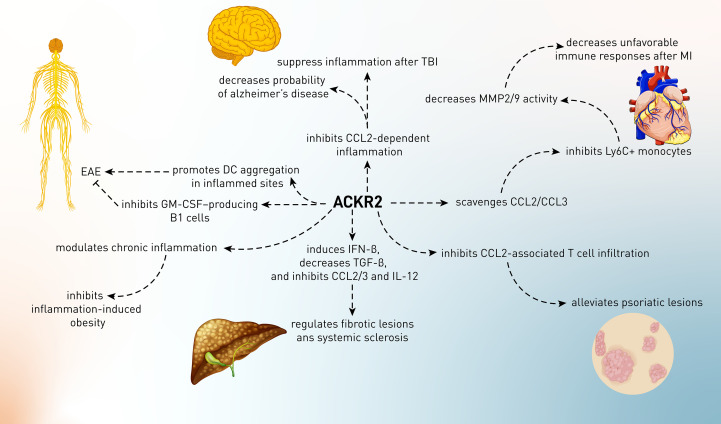
The role of ACKR2 in several conditions including myocardial ischemia, neurological diseases, fibrotic phenomena, and autoimmune disorders such as psoriasis.

Cardiovascular diseases (CVDs) include a wide range of conditions that affect the blood vessels and heart and may present themselves in life-threatening events such as myocardial infarction (MI), aneurysm, and stroke. Myocardial infarction (MI) is histologically characterized by the infiltration of leukocytes, namely, neutrophils (within the first 24 h) and Ly6C^high^ (within 4 days and in the acute phase) and Ly6C^low^ (after acute phase) subsets of monocytes in ischemic zones, and excessive infiltration of innate immune cells has been demonstrated to induce unfavorable remodeling of the left ventricle and heart rupture. The recruitment of inflammatory cells to the ischemic heart is mainly dependent on the CC chemokine members, namely, CCL2, CCL5, and CCL3 and their receptors, CCR2. ACKR2 was shown to be expressed in infarcted myocardium in humans and mice (85, 86). ACKR2 deficiency resulted in elevated chemokine CCL2 and CCL3 levels in the ischemic heart of mice subjected to myocardial infarction. Elevated infiltration of pathogenic neutrophils and Ly6C^high^ monocytes was seen in ACKR2-deficient infarcts, which was associated with increased MMP-2 and MMP-9 activity in the ischemic heart. After MI, ACKR2-deficient animals had a ventricular rupture, and functional analysis indicated that their hearts exhibited symptoms of unfavorable remodeling, including left ventricle dilatation and decreased ejection fraction (**Figure 3**). In a bone marrow chimera experiment, it has been demonstrated that leukocyte-borne ACKR2 had no function in this scenario and that absence of the leukocyte-specific chemokine CCR2 reversed the ACKR2-deficient mice’s unfavorable phenotype. In fact, CCR2 signaling promotes adverse remodeling in myocardial infarction, which was reversed upon ACKR2 upregulation, indicating that the scavenging of CC chemokines by ACKR2 regulates tissue homeostasis and favorable cardiac remodeling after MI and limits pathogenic inflammation (87).

Other than MI, recent research has found that pro-inflammatory molecules are overexpressed in the nucleus tractus solitarii (NTS) of spontaneously hypertensive rats (SHR) when compared to normotensive rats (Wistar–Kyoto rats: WKY), implying that the NTS of SHR may be inflamed abnormally. Therefore, the expression of inflammatory chemokines and cytokines might be involved in the formation of hypertensive phenotype in rats (88). Regarding the involvement of ACKR2 in NTS of SHR, there are no published data. However, it is presented in an oral presentation abstract that ACKR2-deficient mice had lower T-cell content and lower CCR5 expression in the perivascular adipose tissue, which is linked to hypertension. Independent of blood pressure modulation, this is accompanied with enhanced vascular function and reduced ROS generation (89). Therefore, it is needed to explore the role of ACKR2 in hypertension and its association with dysregulation of other chemokine receptors and inflammatory mediators.

### 4.3 Neurological Diseases

#### 4.3.1 Alzheimer’s Disease

AD is the most frequent neurodegenerative condition in the aged population and is the leading cause of dementia in this generation. AD is defined pathologically by the accumulation of amyloid-beta (Aβ) peptides in the gray matter, resulting in insoluble aggregated plaques that are considered to cause neurodegeneration and cognitive impairment. CCL2 is involved in the regulation of monocyte and dendritic cell migration across the blood–brain barrier, as well as the differentiation and migration of macrophages. CCL2 is mostly released by astrocytes and microglia in the brain, with only trace amounts secreted by endothelial cells. CCL2 is produced in the central nervous system (CNS) due to an immunological response to infection, damage, or inflammatory reaction in the CNS (90).

In the animal model of AD, CCL2 is essential for causing chronic inflammation and activation of immune cells, as well as the release of other chemokines. The overexpression of CCL2 causes activated microglia cells to remain in close proximity to the inflamed location. This leads to enhanced activated microglia and elevation of key features of AD, such as amyloid plaques, plaque aggregation, and cognitive loss (91). In AD murine models, inhibition or removal of CCL2 results in decreased amyloid pathology production. These investigations reveal that adequate function and modulation of CCL2 are required for the preservation of innate immune responses in the brain and cognitive function (92).

Cells of the microglia and astrocytes predominate in the CNS when it comes to ACKR2 expression (93, 94). Following CCL2 activation, p-cofilin expression was significantly decreased in cells expressing the ACKR2-V41A receptor compared to wild-type cells. This finding is consistent with the results of an experiment that measured ACKR2 receptor levels following CCL2 stimulation during a 72-h period. Since low levels of p-cofilin in ACKR2-V41A-expressing cells were shown, it was proposed that decreased scavenging activity of ACKR2 is responsible for CCL2 function in the AD (12). However, the action of wild-type ACKR2 in AD has not yet been elucidated, and it was perceived that the function of ACKR2 in AD was just from the hypoactivity of ACKR2-V41A in AD (**Figure 3**).

#### 4.3.2 Traumatic Brain Injury

Other than AD, inflammatory responses are also involved in the pathophysiology of traumatic brain injury (TBI). Inflammation is a natural reaction to trauma and has neuroprotective and regenerative effects because it enables the clearance of cellular debris and the release of neurotrophic substances (95, 96). The secretion of immune mediators in the damaged brain, on the other hand, is hypothesized to contribute to secondary injury *via* the production of reactive oxygen species, nitric oxide, and glutamate from infiltrating immune cells and brain-resident microglia (95, 97). Furthermore, after a TBI, the accumulation of activated microglia and leukocytes in the damaged region is known to promote tissue damage. Chemokines have been considered the primary inducer of the migration of these cells into the injured sites. CCL2 levels are significantly elevated in various TBI models, such as diffuse axonal damage, stab wounds, and lateral fluid percussion (98–100). Furthermore, in a closed head injury (CHI) model of TBI, it has been recently demonstrated that CCL2 is clearly enhanced post-injury and precedes macrophage buildup surrounding the lesion (98, 100). Since the expression of ACKR2 in the CNS has been proven, it is proposed that this receptor might contribute to TBI (93, 94). Following TBI in mice, there was a considerable and progressive elevation of *CCL2* mRNA; thus, the more time passed, the more ACKR2 was increased. This elevation was correlated to the enhancement of CCL2 expression in these mice, and there was a positive correlation between ACKR2 mRNA and survival time after TBI (**Figure 3**). Moreover, in CHI-subjected ACKR2-knockout mice, the rate of mortality and lesion volume was increased, indicating the critical role of ACKR2 in brain responses to injury. Although the underlying mechanism of ACKR2-dependent alleviation of brain injury has not been defined, it is proposed that this function possibly relies on the CCL2’s sequestering and scavenging nature of ACKR2 to suppress deleterious chronic inflammation in the brain (93).

#### 4.3.3 Multiple Sclerosis and Experimental Autoimmune Encephalomyelitis

In contrast to the above-mentioned neurological disorders, ACKR2 has a conflicting role in the murine model of multiple sclerosis MS, i.e., EAE. It was shown that myelin oligodendroglial glycoprotein (MOG) peptide-induced ACKR2^+^ mice compared to ACKR2-knockout mice decrease inflammation and demyelination of the spinal cord and have a lower incidence and severity of EAE episodes (101). The administration of MOG to the ACKR2-deleted mice led to lesser type I cytokines, e.g., IFN-γ production, impaired T-cell priming, or antigen presentation than wild-type models. Thus, the absence of ACKR2 affected the microenvironment required for efficient antigen presentation, leading to defects in T-cell induction. It is yet unclear how the presence of ACKR2 impacts T-cell activation. By discovering CD11c^+^ DC aggregates in ACKR2^–/–^ mice in MOG-inoculation sites, an explanation for the EAE-promoting effects of ACKR2 is that DCs are trapped in the inflammatory sites and do not exit from those sites, leading to an exacerbation of inflammation (101). In contrast, the EAE-suppressing role of ACKR2 is attributed to innate-like B cells, which are one of the ACKR2-expressing cells in the body (19). It has been shown that in the absence of ACKR2, B1 cells migrate towards CXCL13 (but not in an ACKR2 ligand-dependent manner) (**Figure 3**). These B1 cells produce GM-CSF, a growth factor for Th17, and enhance EAE-promoting Th17 cells. Therefore, during the induction of neuropathic autoimmunity in mice, ACKR2 can regulate GM-CSF-producing B cells and T-cell priming, and its deletion causes subtle changes in disease development (102). Finally, it is proposed that future studies should focus on the concept of remote control of immune response and the transfer of chemokines to the inflammatory sites since chemokines can be produced in one site and then may be transferred to other similar sites through draining into lymph nodes (103). Given ACKR2’s regulatory role in inflammation and T-cell priming, it is critical that studies are now being conducted to explore further the expression and regulation of ACKR2 in the draining lymph nodes and inflamed tissues of patients with autoimmune disease. It remains to be understood whether ACKR2-mediated chemokine scavenging can influence disease progression in humans. A better knowledge of these aspects of ACKR2 biology might lead to the development of novel therapeutics that exploit the artificial increase/decrease of ACKR2 in chronically inflamed tissues to decrease chemokine-driven inflammation.

Regarding the human counterpart of EAE, i.e., MS, it has been shown that ACKR2 mRNA expression in PBMC derived from relapsing-remitting MS subjects was decreased. However, no link was found between this receptor and other cytokines implicated in the pathogenesis of MS, such as TNF-α and IL-33 (104). Therefore, it is necessary to elucidate the levels of ACKR2 in the different stages of MS and their correlation with the disease manifestation. Moreover, the role of ACKR2 downregulation should be clarified in MS pathogenesis, and the levels of inflammatory chemokines also need to be measured.

### 4.4 Autoimmunity and Inflammatory Situations

#### 4.4.1 Psoriasis

To begin the ACKR2 story in human psoriasis, it was shown that this receptor was downregulated in psoriatic tissue but significantly upregulated in the uninvolved skin of psoriatic patients, particularly in the epidermis at the mRNA and protein levels. However, inflammatory or T-cell-recruiting chemokines such as CCL2 were found in the epidermis of psoriatic plaques and notably in unaffected psoriatic skin (but not in healthy controls’ skin), indicating the inflammation-regulatory role of ACKR2 at distant sites (105). This research demonstrated that ACKR2 expression was enhanced in normal skin compared to psoriatic plaques in psoriatic patients. Although ACKR2 was upregulated in certain organs (heart and liver) in topically imiquimod-treated mice, it was not upregulated in other skin regions. The lack of ACKR2 upregulation at distant skin spots is puzzling since the imiquimod-only-treated mice lost weight systemically. ACKR2 was also downregulated in imiquimod-treated spots, like in humans. The most intriguing findings came from a test to see if inducing psoriatic inflammation in one spot on the skin, followed by IFN-γ injection, enabled mice to “protect” themselves against further cutaneous inflammation when treated with imiquimod. To find this out, mice were treated at the primary location with and without systemic IFN-γ. Results showed that IFN-γ therapy reduced the skin’s capacity to react to imiquimod at the primary location. This was followed by a reduction in the frequency of T cells in the epidermis and decreased inflammation at the distant site after systemic IFN-γ and topical imiquimod therapy (**Figure 3**). Collectively, distant areas generated less inflammation from the primary location of psoriasis and after systemic IFN-γ therapy. The protection of distant sites seems to need ACKR2, since it was not seen in *ACKR2*^–/–^ mice under identical settings, but what was evoked at primary sites (probably not only IFN-γ) that increases ACKR2 expression at distant locations is still unknown (105–107).

Regarding the molecular mechanism of the downregulation of ACKR2 in psoriatic sites, it has been shown that psoriatic-associated microRNAs play a pivotal role. In keratinocytes and LECs, it was found that miR-146b and miR-10b bind directly to the 3’-UTR of *ACKR2* gene and decrease ACKR2 mRNA and protein levels in HEK293 cells. ACKR2 expression is further downregulated in psoriasis patients after cell damage, which is a key trigger for the creation of new plaques (also called the Koebner phenomenon). When cells are subjected to tensile stress, ACKR2 is rapidly downregulated, and miR-146b is simultaneously upregulated (31). Collectively, it is proposed that employing a mechanism in which ACKR2-suppressive miRNAs in the psoriatic lesion are targeted might be promising in future treatment.

#### 4.4.2 Fibrosis

Fibrosis, sometimes referred to as fibrotic scarring, is a pathological process of wound healing in which connective tissue overtakes normal parenchymal tissue, resulting in significant tissue remodeling and the development of everlasting scar tissue. Repeated injuries, chronic inflammation, and healing are all prone to fibrosis, which occurs when fibroblasts create an abnormally large amount of ECM components, such as collagen, resulting in the formation of a permanent fibrotic scar (108, 109). Uncontrolled inflammation may lead to fibrosis, which is characterized by excessive collagen deposition by myofibroblasts during tissue remodeling, resulting in tissue thickening. Therefore, in order to achieve proper wound healing and limit excessive fibrotic scarring, resolution of inflammation is inevitable. The differentiation of infiltrating inflammatory monocytes into reparative phagocytic macrophages takes place during the resolution of inflammation (110, 111). On the other hand, these macrophages have the potential to cause tissue fibrosis if they are not regulated in a timely manner. The production of ECM proteins during tissue repair and inflammation resolution is primarily mediated by fibroblasts that develop into active myofibroblasts. ECM proteins include collagen subtypes, laminin, fibronectin, and other related proteins (112, 113).

An antiviral cytokine that is also linked to lupus is called IFN-β, which belongs to type I interferons (114). However, recently, it has been found that after reprogramming macrophages to be pro-resolving non-phagocytic ones, they produce and secrete a lot of IFN-β, which boosts some pro-resolving events (114). IFN-β, for example, inhibits the release of pro-inflammatory cytokines and boosts the production of anti-inflammatory and anti-fibrotic IL-10 from macrophages that are in the “resolution phase.” IFN-β also inhibits lung fibrosis caused by bleomycin by cutting down on tissue TGFβ and thrombospondin, and the IFN-β/IL-1 receptor antagonist axis plays a role in the TLR5-induced improvement of liver fibrosis (**Figure 3**) (115, 116).

Since ACKR2 is a pro-resolving chemokine receptor, its role in skin fibrosis has been studied. There was a rise in epidermal and dermal thickness, atrophy of the subcutaneous adipose tissue, intensified disorientation of collagen deposition, and enhanced distortion and loss of hair follicles in fibrotic skin lesions from *ACKR2*^–/–^ mice. *ACKR2*^–/–^ mice also had lower amounts of IFN-β and IL-10 but higher levels of CCL2 and CCL3, the pro-fibrotic cytokine TGF-β, and the immune-stimulating cytokine IL-12 in their impacted skin areas. Exogenous IFN-β therapy reversed, at least in part, all of the pro-fibrotic outcomes and lesion size in *ACKR2*^–/–^ mice and enhanced the expression of lipoxygenase (a key effector in the resolution of inflammation by generating pro-resolving lipid mediators and promoting apoptotic cell efferocytosis) in both *ACKR2*^–/–^ and WT animals. After bleomycin exposure, *Ifnb*^–/–^ mice showed increased pro-fibrotic indices. There is evidence to indicate that ACKR2 is one of the key players in preventing chronic inflammation-induced skin scarring caused by promoting IFN-β production (117).

##### 4.4.2.1 Pulmonary Fibrosis

Idiopathic pulmonary fibrosis (IPF) is a lethal disorder resulting from aberrant fibroblast and immune system activation, which leads to irreversible fibrotic changes in the lungs. This disorder like fibrosis in other tissues is caused by excessive immune responses and production of chemokines such as CCL2, CCL3, CCL11, and CXCL1–3 in the lungs, leading to initiating the inflammatory reactions and inappropriate activation of fibroblast. Although ACKR2 has been implicated as a chemokine scavenging receptor to reduce inflammatory responses in several tissues, it has been shown that ACKR2 plays a promoting role in IPF. In bleomycin-induced IPF in *ACKR2*^–/–^ mice, it has been confirmed that these mice displayed a reduced pulmonary dysfunction and fibrosis that were related to decreased expression of genes associated with fibrogenesis and Th2/Th17 responses. ACKR2 knockdown increased IFN-γ expression (an antifibrotic cytokine and a negative regulator of TGF-β1). Moreover, downregulation of ACKR2 increased the recruitment and influx of CCR2^+^/CCR5^+^ T_γδ_ cells (anti-fibrotic and homeostatic T cells) in the lungs, which resulted from upregulation of CCL5, CCL12, and IFNγ. In conclusion, ACKR2 is activated during bleomycin-induced pulmonary fibrosis, and its deletion reduces mortality, lung damage, inflammation, and fibrosis. ACKR2 is a determinant for managing the recruitment of CCR2^+^/CCR5^+^ IFN-γ–producing T_γδ_ cells, which generate IFN-γ and regulate the development of the Th17 response and fibrosis intensity (118).

#### 4.4.3 Obesity

Obesity is the outcome of a persistent anabolic state defined by increased caloric intake, decreased energy expenditure, or both (119). Experiments have shown that dietary factors, notably saturated fatty acids, induce inflammatory responses in the hypothalamus, impairing the function of essential neurons involved in regulating whole-body energy status (120). Numerous investigations have shown that resident microglia and astrocytes are involved in this inflammatory process as cellular components (121). The concept that peripheral immune cells may be attracted to the hypothalamus in response to hypothalamic inflammation and chemokines is now under active examination (121, 122). The chemokine CX3CL1 (fractalkine) is quickly produced in the hypothalamus after the introduction of a high-fat diet (HFD) and functions as a mediator for the migration of bone marrow-derived cells to augment the hypothalamic microglia-induced inflammation. The hypothalamus was anticipated to produce chemokines other than fractalkine during the early stages of diet-induced obesity, and these chemokines may have a role in the metabolic consequences associated with obesity (123). To find this, the expression pattern of chemokine and its receptors was analyzed in the hypothalamus of HFD mice to compare obesity-prone (OP) and obesity-resistant (OR) mice. Results showed that only 3 days after the introduction of an HFD, Ackr2 was shown to be one of the most drastically decreased transcripts in the hypothalamus of OP mice compared to OR ones. Improved hypothalamic inflammation and reduced obesity-related glucose intolerance were achieved by hypothalamic lentiviral overexpression of ACKR2. It is therefore hypothesized that increased expression of ACKR2 in the hypothalamus lowers diet-induced inflammation, which in turn improves glucose tolerance in obesity-prone mice. Thus, ACKR2 appears as a novel immunomodulatory actor in the setting of diet-induced inflammation of the hypothalamus, and approaches aimed at raising ACKR2 hypothalamic expression may be practical for enhancing metabolic control in obesity (124). However, there is a lack of examination of inflammatory CC chemokines, which needs to be evaluated.

#### 4.4.4 Rheumatic Diseases

Rheumatic disorders, often known as rheumatism, are a group of diseases that affect the connective tissues, especially the joints, causing chronic and often intermittent pain. Rheumatism is a general term that refers to a wide range of diseases, including arthritis and non-articular rheumatism, including spondyloarthropathies, lupus, Sjogren’s syndrome, systemic sclerosis, and sarcoidosis.

Systemic scleroderma, also known as systemic sclerosis (SSC), is an autoimmune rheumatic condition characterized by excessive collagen production and deposition in the skin and internal organs, as well as injury to small arteries, which is collectively called fibrosis (125). Therefore, vasculopathy, abnormal immune system activation, and multiorgan fibrotic alterations caused by excessive ECM deposition are all considered SSC symptoms (125). Chemokines and their receptors have received much attention in assessing SSC pathogenesis due to the evident participation of inflammatory processes in SSC pathology (126). Chemokines, in particular CCL2, CCL5, CCL7, and CXCL8, have been implicated in initiating and maintaining abnormal fibroblast activation and the accompanying ECM deposition that characterizes this condition (127–129). Since the impact of chemokines, leukocyte accumulation, and inflammatory responses have been identified in the pathogenesis of SSC, the role of ACKR2 should be important in SSC. Analysis of ACKR2 expression in PBMCs of SSC patients and serum levels of this receptor in these patients has shown that ACKR2 levels were increased in the serum, platelet-rich plasma (PRP), and PBMCs (mRNA and protein) of patients compared to the normal group. A negative association was observed between the *ACKR2* mRNA and a lack of increase in serum levels of CCL2, CCL3 (weakly), and CCL4 in patients with SSC compared with healthy control, indicating that ACKR2 might be a response to alleviate the SSC pathogenesis (130). In contrast to this study, no differences in ACKR2 mRNA levels were found between pregnant SSC subjects and healthy pregnant women in another study (131). A possible explanation for this difference between these two studies could be that samples were driven from pregnant women in the second study, and the levels of ACKR2 are commonly increased in pregnancy. However, an in-depth investigation is required to identify how these findings of systemic elevation of proinflammatory chemokines and ACKR2 in serum and PBMC connect to peripheral disease involvement, such as skin and lung, which clearly demonstrate an active and unregulated immune response in SSC. Therefore, inflammatory chemokines and ACKR2 have emerged as attractive candidates for future exploration and possible therapeutic intervention in these investigations. In SSC, where the treatment choices are restricted, this is particularly important because end organ damage, which reduces the quality of life and increases mortality, cannot be prevented.

#### 4.4.5 Transplantation and Graft-Versus-Host Disease

Corneal allograft rejection is mostly mediated indirectly by allorecognition, in which newly recruited host antigen-presenting cells (APCs) process and present corneal alloantigens to naive host T cells. The induction of an allospecific Th1 response then facilitates corneal transplant rejection. Syngeneic corneal transplants done in naive hosts, in contrast to allografts, are accepted forever. Regardless of the result, both corneal syngeneic and allogeneic transplants stimulate corneal hem- and lymphangiogenesis, which is thought to have a substantial impact on the graft’s outcome or the effectiveness of a subsequent graft (132, 133). ACKR2 deletion was associated considerably with increased rejection of syngeneic corneal grafts, in which almost 60% of ACKR2^–/–^ mice rejected their syngrafts 1 week after surgery. This is a surprising finding, given that ACKR2^–/–^mice are completely histocompatible with WT littermate mice and form such syngeneic graft rejection. However, the impact was attributed to an enhanced innate immune response in ACKR2^–/–^ mice, indicating the participation of damage-associated molecular patterns (DAMPs) under the sterile circumstances of transplant surgery. Specifically, it was claimed that DC-expressed ACKR2 contributes to DC behavior modification by increasing maturation and allosensitization. Despite the reduced allospecific T-cell response shown in ACKR2^–/–^ mice, no difference in allograft survival was observed between WT and ACKR2^–/–^ mice, suggesting that any such impact was limited to syngeneic grafts (134). The alternative suggested role of ACKR2 in allograft rejection was attributed to its role in the development of lymphatic vessels and lymphangiogenesis (135). Syngeneic grafts, but not allogeneic grafts, increased lymphatic sprouting and infiltration of Lyve-1^+^ cells (LEC cells) in ACKR2^–/–^ mice at the early post-graft stage, although lymphatic density was comparable to that of WT grafted mice. It was proposed that deletion of ACKR2 increases the proximity of pro-lymphangiogenic macrophages to LECs. Therefore, it was proposed that ACKR2, through modulating lymphangiogenesis, might be involved in the suppression of corneal allograft rejection (135).

GVHD (graft-versus-host disease) is the most serious clinical issue that may occur following allogeneic BM cell transplantation. GVHD’s target tissues are invaded by T cells, and it has been shown that local chemokine synthesis is considered a mediator of this invasion (136). It is noteworthy that there is a quick and temporary rise in chemokine production in GVHD target tissues prior to T-cell infiltration, and that increase is enhanced by the allogeneic response (136). Inhibition of inflammatory chemokines has been linked to reduced GVHD mortality, and chemokines have been identified as molecular targets for the therapy of acute GVHD. In mice, Ly6C^high^/Gr1^+^ myeloid cells, which express CCR2, exert immunomodulatory and immunoregulatory functions and recruit into tissues whereby CCL2 is produced (137). Therefore, it was proposed that the increase in the population of recruited Ly6C^high^/Gr1^+^ myeloid cells into the grafted bone marrow might be beneficial for the suppression of GVHD. Regarding the involvement and effect of ACKR2 expression in GVHD, it has been shown that the levels of Ly6C^high^/Gr1^+^ monocytes have been increased CCR2/CCL2-dependently in the bloodstream and spleen of ACKR2^–/–^ mice. These bone marrow-accumulated monocytes displayed immunosuppressive functions by producing arginase-1 and cyclooxygenase-2, leading to reduced T-cell priming and suppression of the inflammatory milieu in the bone marrow (138). In conclusion, CCR2-dependent trafficking of monocyte subsets is regulated by ACKR2, according to the findings provided in this study. ACKR2 controls the immunosuppressive phenotype acquired by Ly6C^high^ myeloid cells, and its lack provides partial protection against GVHD. It is proposed that ACKR2 inhibitors might be used in conjunction with standard treatments to slow down the course of GVHD. ACKR2 inhibition may also be effective in the development of novel treatment strategies for GVHD patients, particularly steroid-resistant patients, that depend on the transfer and/or increase of immunosuppressive and regulatory leukocyte populations.

#### 4.4.6 Kidney Inflammatory Conditions

Acute renal injury and glomerulonephritis are two major inflammatory responses that are considered risk factors for the development of chronic kidney diseases and renal failure (139, 140). In acute kidney injury (AKI), the loss of nephrons owing to inadequate recovery and fibrotic tissue remodeling is the cause of chronic renal damage. After initial damage, the inflammatory response determines the AKI outcome. AKI caused by ischemia-reperfusion injury (IRI) promotes an influx of leukocytes into the damaged tubules, further mediating tubular damage. In addition, chronic inflammation stimulates phagocytes, fibroblasts, and myofibroblasts in the renal interstitium. Their excessive ECM formation not only replaces permanently injured nephrons but may also contribute to progressive tubular injury by impairing capillary blood flow and oxygen and nutrient transport. Limiting renal inflammation following AKI prevents tubular damage and renal fibrosis (i.e., the progression from AKI to CKD). Following IRI, the inflammation subsides, allowing tubular regeneration to occur, while persistent inflammatory damage caused by infiltrating leukocytes results in nephron loss and renal fibrosis, which are characteristic of chronic kidney disease and require dialysis. *In vitro* studies have shown that ACKR2 reduces the amount of leukocyte infiltration, inflammation, and fibrotic tissue remodeling that occurs following renal IRI, hence avoiding the development of chronic kidney disease (139). *In vitro*, Ackr2 loss results in increased CCL2 levels in TNF-stimulated tubulointerstitial tissue when compared to the wild type. In Ackr2-deficient mice with early IRI, 1 or 5 days after transient renal pedicle clamping, tubular damage was comparable to that seen in wild-type mice, while the concentration of mononuclear phagocytes was greater in postischemic Ackr2^–/–^ kidneys than in wild-type mice. The long-term results of the study revealed that Ackr2-deficient kidneys had more serious tubular damage 5 weeks after IRI, which was linked with consistently elevated renal infiltrates of mononuclear phagocytes, T cells, Ly6C^high^ inflammatory macrophages, and inflammation (139). The lack of Ackr2 in the kidneys 5 weeks after IRI resulted in a significant deterioration of renal fibrosis, as shown by increased expression of matrix molecules, renal accumulation of α-SMA^+^ myofibroblasts, and BM-derived fibrocytes in the kidneys of those mice (139).

The function of ACKR2 was also examined in another inflammatory condition, i.e., glomerulonephritis. As a prominent cause of renal failure globally, glomerulonephritis eventually leads to end-stage renal disease. Typically, it is induced by glomerular deposition or *in situ* production of immune complexes, which in turn elicit adaptive cellular and humoral immune responses to native or planted glomerular antigens, infiltration of renal leukocytes, and cell-mediated renal damage. To investigate the possible anti-inflammatory activities of ACKR2 in glomerulonephritis, autologous nephrotoxic nephritis was induced in C57BL6 and Ackr2-deficient mice. Interstitial lymphatic endothelium was shown to be the primary site of ACKR2 expression in the kidneys during nephritis. An increase in albuminuria and urea concentrations was seen at two weeks in Ackr2^–/–^ mice compared to wild-type littermates (140). The histological investigation found increased structural damage in the glomerular and tubulointerstitial compartments of the Ackr2^–/–^ kidneys. CD4^+^ T lymphocytes and mononuclear phagocytes accumulated in the tubulointerstitial area but not in the glomeruli of ACKR2-knockout mice. Ackr2^–/–^ kidneys showed increased expression of inflammatory mediators, including markers of fibrotic tissue remodeling (enhanced myofibroblast accumulation plus upregulation of fibronectin, laminin, procollagen 1, and procollagen 4), as well as greater levels of inflammatory CCL2 (140). The deficiency of Ackr2 in tubulointerstitial tissue but not glomeruli enhanced the concentration of chemokines *in vitro*. According to these findings, ACKR2 is expressed in interstitial LECs, ensuring the efflux of activated leukocytes into local lymph nodes (140). Furthermore, nephritic Ackr2^–/–^ mice had diminished adaptive cellular immune responses, as seen by decreased regional T-cell activation in their kidneys. However, this did not prevent the development of exacerbated damage in the kidneys of Ackr2^–/–^ mice suffering from nephrotoxic nephritis as a result of elevated tubulointerstitial chemokine levels, leukocyte infiltration, and fibrosis occurring at the same time. Collectively, the role of ACKR2 in controlling renal inflammation and fibrotic remodeling in progressive nephrotoxic nephritis has been established. As a result, ACKR2 is considered a chemokine PAKMAN, which may serve as a therapeutic target in the treatment of immune complex glomerulonephritis (140). These therapies may involve improved systemic scavenging of proinflammatory chemokines through ACKR2-IgG fusion proteins, equivalent to etanercept-based TNF inhibition. More ingeniously, renal ACKR2 expression may be increased by precisely targeting the renal tubulointerstitium with mRNA-stabilizing agents.

#### 4.4.7 Colitis

As mentioned in the tumor section, the absence of ACKR2 in mice can lead to colitis-associated colon cancer (59, 60). Several studies confirmed the accumulation of inflammatory mediators including CCL2, 3, 4, 5, 7, and 8 in the colonic microenvironment and mucosa. In terms of ACKR2, it has been shown that the expression of this decoy receptor has upregulated during the induction and development of colitis with dextran sodium sulfate (DSS) in mice. Although ACKR2-deficient mice have shown reduced tissue damage in DSS-induced acute colitis, they showed a substantial increase in the production of IFN-γ and IL-17A, and an increased number of IL-17A-secreting T_γδ_ cells in the lamina propria. Moreover, ACKR2 had no effect on the release of chemokines from inflamed colons. IL-17A has been shown to have protective effects on DSS-induced colitis by showing antimicrobial properties, activating neutrophil recruitment, enhancing intestinal epithelial integrity, and inducing cytoprotective prostaglandins. Therefore, recruitment of IL-17A-secreting T_γδ_ cells protects mice against DSS-induced colitis. ACKR2 expression in colitis mucosa prevents migration of IL-17A-secreting T_γδ_ cells to this microenvironment, leading to exacerbation of colitis in mice (141). The results of this study and its contradiction with the outcomes of ACKR2 involvement in colitis-associated colon cancer suggests that ACKR2 effects on different conditions might be varied due to the contribution of several other factors. Therefore, a simultaneous comparison of these two conditions might broaden our knowledge of the progression of colitis and colitis-associated colon cancer.

#### 4.4.8 Chronic Obstructive Pulmonary Disease and Asthma

The analysis of ACKR2 expression in COPD patients showed a different pattern of expression. Results showed that ACKR2 was mainly expressed on alveolar macrophages but not on lymphatic vessels in COPD patients. In terms of intensity of expression, COPD patients had significantly increased ACKR2 expression in alveolar macrophages compared to controls, which was positively correlated to the severity of the disease. Moreover, ACKR2 expression was negatively correlated to lung function parameters and the levels of inflammatory cytokine TNF-α and IL-32. The elevation of ACKR2 expression in alveolar macrophages of COPD patients indicated its inflammation-promoting functions in COPD patients, which leads to immune cells’ (CD8^+^ cells) activation in the lungs and might promote COPD progression (142). However, it is thought that the role of ACKR2 in COPD should be explained in terms of how it works.

In asthma, like other pulmonary diseases including COPD and IPF, the expression of ACKR2 has also been dysregulated. It has been shown that the promoter of the ACKR2 gene was methylated in the CpG region in the remission phage patients (143). The effectiveness of ACKR2 to reduce chemokine levels in the lungs depended on the chemokine concentration. CCL17 and CCL22 were prevalent in the airway, and when they were within a certain concentration range, ACKR2 reduced their levels. CCL3, CCL5, and CCL11 airway concentrations, on the other hand, were low and unaffected by ACKR2. ACKR2-deficient mice exposed to allergens showed more DCs, T cells, and eosinophils in the lung parenchyma, as well as more eosinophils in the airway, than WT C57BL/6 mice. Moreover, ACKR2-deficient animals displayed lower airway responses to methacholine than C57BL/6 mice. As a result, ACKR2 possesses conflicting effects on asthma in which it has anti-inflammatory properties while promoting reactions in the airways. In conclusion, ACKR2 deficiency promotes inflammation while decreasing airway reactivity. These results imply that suppressing the activity of ACKR2 might be a unique method to reduce allergic asthmatic airway responses (144).

### 4.5 Infections

Sepsis is a potentially lethal organ dysfunction induced by an uncontrolled host response to infection. By recruiting neutrophils to an infected site, the infectious agents are attacked, and the pathogen is prevented from spreading into the circulation. However, chemokine-mediated neutrophil infiltration into vital organs leads to organ failure and, ultimately, mortality during sepsis (145–147). Using a cecal ligation and puncture (CLP, the most frequently used model for inducing sepsis) murine model, it was demonstrated that ACKR2-deficient mice had a significantly lower survival rate compared with similarly treated WT mice (148). However, bacterial load and neutrophil infiltration into the peritoneal cavity were similar between ACKR2-deficient and WT mice during the experiment. When compared to WT mice, ACKR2^–/–^ mice had more neutrophil infiltration and higher levels of inflammatory CC chemokine in the lung, heart, and kidney, indicating that ACKR2 regulates neutrophil infiltration and chemokine accumulation in secondary organs during sepsis. *ACKR2*^–/–^ mice also had more severe lung and kidney lesions than WT mice (148). In line with these findings, WT mice with non-severe sepsis showed greater levels of ACKR2 expression in the lungs, heart, and kidney than WT mice with severe sepsis. In addition to murine models, analysis of ACKR2^+^ cells in the lungs of septic patients revealed a substantial increase in the number of these cells expressing ACKR2^+^ compared to non-septic controls, suggesting that the elevated level of ACKR2 in septic patients is a protective response in sepsis, and a lack of ACKR2 results in increased chemokine production, neutrophil accumulation, and damage to vital organs (148).

#### 4.5.1 Mycobacterium tuberculosis

An important part of host defense against *Mycobacterium tuberculosis* (Mtb) is the formation of granulomas. Mycobacteria-reactive T cells, especially IFN-γ-secreting ones, play a critical role in this process (149). In order to generate granulomas, lymphocytes must be activated, recruited to the infection site, migrated into the tissues, and positioned around mycobacteria-infected macrophages in order to form granulomas. This colocalization makes it easier for T-cell-derived cytokines to activate anti-bacterial mechanisms in infected macrophages (149). However, certain mycobacteria live inside macrophages, causing chronic granuloma development with the accumulation of infected macrophages, epithelioid cells, and T cells. In addition to containing the mycobacterial infection and preventing organ spread, these granulomas cause unrestricted chronic inflammation and lung immunopathology by displacing granulomas and destroying parenchymal tissue (150, 151). In most situations, following tissue healing, inflammation diminishes, and the tissue recovers to its homeostatic state. Ineffective inflammation resolution may lead to persistent inflammatory processes (150, 151). It is assumed that the immunological response to Mtb is dependent on the production of chemokines. Infection of murine bone marrow-derived macrophages with Mtb leads to the production of TNF-α, as well as various chemokines, including ligands of CXCR3, CCR5, and CCR2, namely, CXCL9, CXCL10, CXCL11, CCL5, and CCL2 (152).

**Figure 4:**
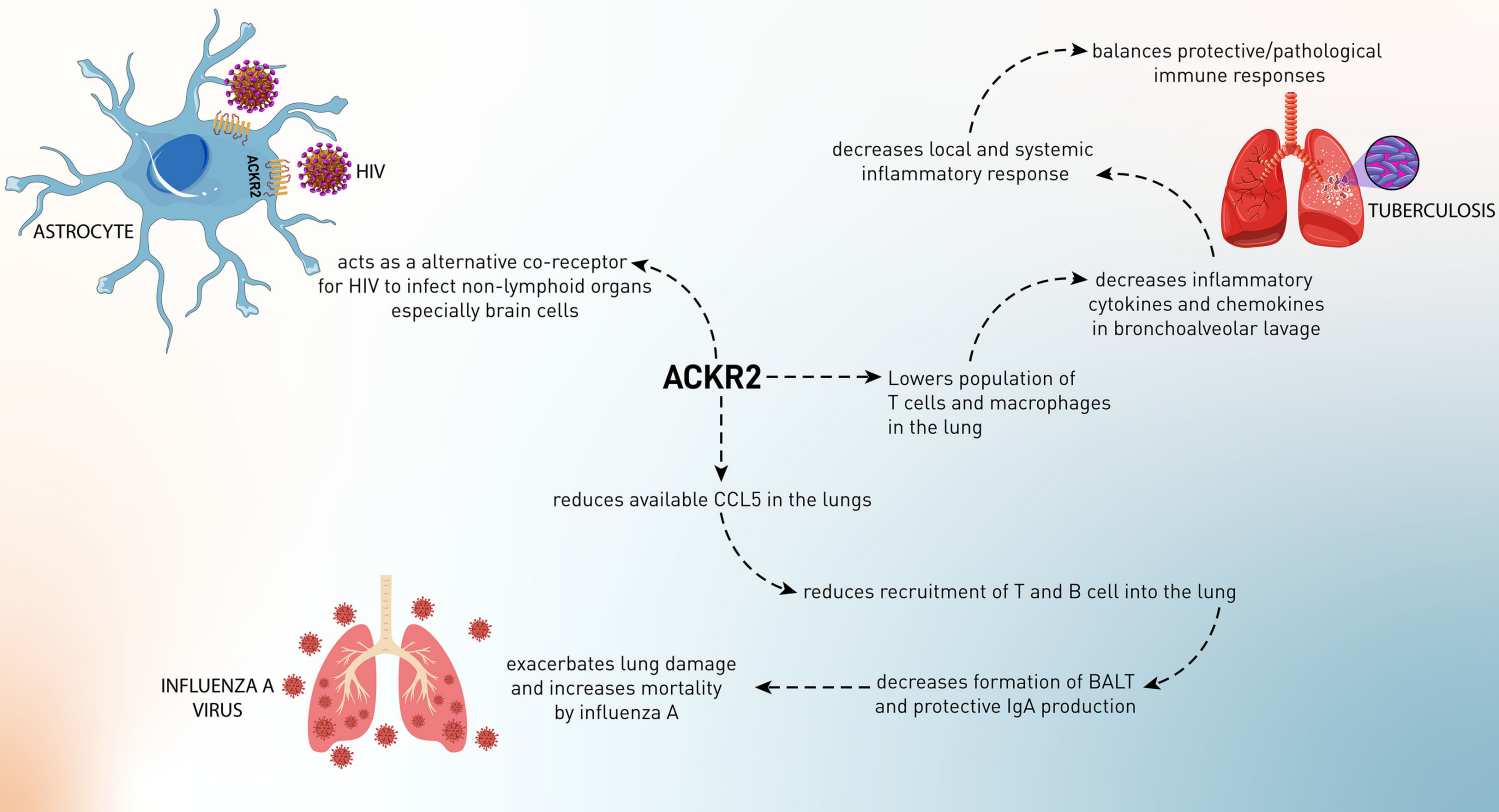
The role of ACKR2 in infections including *Mycobacterium tuberculosis*, HIV, and influenza.

ACKR2 expression was found in LECs and dispersed leukocytes in pulmonary tuberculosis in the lungs and lymph nodes. However, ACKR2 transcripts in the liver, spleen, and lungs were not significantly altered 4, 8, or 12 weeks after the Mtb infection. These findings support recent observations that ACKR2 is mostly expressed in LECs and does not alter much during Mtb infection. Despite insignificant expression of ACKR2 during Mtb infection, it has been shown that Mtb infection increased mortality in Ackr2^–/–^ mice, with 20% dying by week 8, 50% by week 12, and 100% by week 16 (153). In spite of their increased vulnerability to Mtb infection, Ackr2-deficient mice had similar bacterial burdens (**Figure 4**). In Ackr2-deficient mice, at all time points following infection, the population of macrophages, CD4^+^ and CD8^+^ lymphocytes, and other immune cells was considerably more significant in lungs and liver, leading to higher production of IL-1β, IFN-γ, CCL2, CCL3, CCL5, and TNF-α in the bronchoalveolar lavage of infected mice. Therefore, the elevated levels of proinflammatory chemokines and cytokines induced by Mtb infection in ACKR2 deficiency lead to a local and systemic inflammatory response (153). Collectively, ACKR2 has been shown to regulate the production of inflammatory chemokines and cytokines, DC, and T-cell migration, and presumably their maintenance in infected tissues. Thus, ACKR2, by avoiding an excess of chemokines in the inflamed tissue, may encourage the resolution of chronic inflammatory reactions and offer a fine mechanism for balancing protective and pathological immune responses (153).

#### 4.5.2 Human Immunodeficiency Virus

In up to 30% of AIDS patients, HIV infection in the brain causes dementia (154). Microglia, astrocytes, and sometimes neurons are infected by HIV-1 and -2, while the role of cells other than macrophages in brain disease is debatable (154). Other investigations have shown a function for the mannose receptor in astrocyte infection, whereas brain-tropic isolates have been found to need fewer CD4 on the cell surface for effective infection. It has been shown that CCR5^–^/CXCR4^–^ astrocytes are still susceptible to R5 HIV infections (155). Further research revealed that this infection was vulnerable to RANTES, eotaxin, and MIP-1 inhibition, suggesting that a subclass of CC chemokine receptors was implicated (94, 155). Examining the hypothesis that ACKR2 might be a co-receptor for HIV1/HIV2 in the absence of common co-receptors, including CCR5 and CXCR4, showed that ACKR2 may serve as a co-receptor for HIV, and ACKR2-expressing cells are prevalent in primary dual-tropic HIV1 and HIV2 isolates. Moreover, infection of astrocytes with HIV was decreased in the presence of ACKR2 ligands, including RANTES and eotaxin, in high concentrations (94). These observations indicate that HIV1/2, by employing ACKR2, infects brain cells and even other circulating cells, including PBMCs and macrophages, suggesting that ACKR2 acts as an alternative co-receptor for HIV (**Figure 4**) (94, 155). Therefore, using ACKR2 as a co-receptor in HIV infection not only facilitates the dissemination of the virus in the non-lymphoid organs, especially the brain, but also complicates AIDS treatment.

#### 4.5.3 Influenza A Virus

Influenza A virus (IAV)-induced inflammation is a critical process for viral clearance, inducing adaptive immune responses, and getting the lung to a hemostatic status. However, an increased immune response characterized by excessive chemokine production might result in severe lung damage that contributes to death. Certain chemokines are linked to an increase in immunopathology after IAV infection, whereas others are necessary for the virus to be effectively controlled (156, 157). CCL5 is a pleiotropic chemokine that promotes the recruitment, proliferation, and activation of antigen-specific T cells such as CTLs and has been identified as a critical therapeutic target for a variety of infections (158, 159). The expression of ACKR2 in the lungs was raised immediately following IAV infection, which occurred prior to the virus-induced lung dysfunction. Mice that were ACKR2-deficient (ACKR2^–/–^) were protected against IAV, with a reduced viral load and dysfunction of the lungs (160). It is thought that the loss of ACKR2 resulted in increased amounts of CCL5 in the airways, which is released by mononuclear and plasma cells in the lung parenchyma. Increased recruitment of T and B lymphocytes, as well as the formation of inducible BALT (bronchus-associated lymphoid tissue) and the production of IgA in the airways of ACKR2^–/–^ mice following IAV, was observed as a result of the higher chemokine gradient. It was shown that blocking CCL5 in ACKR2^–/–^ mice hindered lymphocyte recruitment, which increased bronchoalveolar lavage fluid protein concentrations and respiratory failure (**Figure 4**) (160). CCR5-deficient mice, on the other hand, had a more severe IAV infection, with an increased number of neutrophils, lung damage, and higher mortality. When IAV infection is present in wild-type mice, results reveal that ACKR2 reduces CCL5 levels, which, in turn, reduces the recruitment of CCR5^+^ Th1, Tregs, and B cells, all of which are important in pathogen control. CCR5 protects against IAV infection by coordinating the innate and adaptive immune responses in mice, whereas ACKR2 is harmful to this process (160).

## 5 Conclusion, Therapeutic Approaches, and Prospective

*In vitro*, *in vivo*, and clinical studies have indicated that ACKR2 is dysregulated in different conditions in the body. Therefore, down- or upregulation of ACKR2 in these conditions prompted investigations to find underlying mechanisms concerning ACKR2’s involvement in diseases. In cancer, ACKR2 has been shown to act as a double-edged sword and play a paradoxical role. In some studies, ACKR2, through inhibiting inflammation, mediates the resolution of inflammation in various conditions such as infections, autoimmune diseases, and cancer. Therefore, using mechanisms that lead to upregulation of ACKR2 might be promising in the treatment of those diseases. For example, it has been shown that some miRNAs, such as miR-146b, can suppress ACKR2 expression. In this regard, targeted modulation of miR-146b through siRNA might be promising in the upregulation of ACKR2 to induce scavenging inflammatory chemokines (31, 32). Another approach for upregulating ACKR2 in inflammatory diseases is using recombinant ACKR2, which can act as a decoy receptor to possibly inhibit the functions of CC chemokines in blocking the infiltration of inflammatory cells into the inflamed tissues.

The physical interaction of proteins, or protein–protein interaction (PPI), that leads to the formation of enormous and densely linked networks is a fascinating area of research. Protein interaction networks effectively develop biological and biomedical applications by abstracting core scientific concepts (161). Protein interactions determine molecular and cellular processes that govern healthy and pathological states in organisms, based on their primary functions in biological function. As a result, such networks support the comprehension of pathogenic (and physiologic) pathways that cause illness onset and progression. Therefore, this information may be used to develop successful diagnostic and treatment procedures (161). Furthermore, the structure and dynamics of protein networks are disrupted in complex disorders such as cancer and autoimmune diseases, according to the findings of various research. Based on this link, a unique paradigm is proposed in order to prove that protein interaction networks, rather than individual molecules, might be the focus of therapy for the treatment of complex multigenic disorders (161). Regarding ACKR2, it is suggested that using this method might elucidate possible ACKR2 interactions with other proteins other than CC chemokine ligands, which can be applied in the treatment of diseases. Moreover, as has been discussed, ACKR2 was dysregulated in several of the mentioned complications. Analyzing this receptor in affected tissue showed a hint to point out a diseased condition. Therefore, examination of ACKR2 expression accompanied by several other biomarkers may give us an opportunity to determine the trend of disease progression.

Most studies regarding ACKR2 function and its mechanism of action in physiological and pathological situations are based on observations extracted from *in vitro* and *in vivo* studies, which are almost animal models. As a result, it is proposed that future investigations can be more humanized to be translated into clinical trials. Finally, it is hoped that using ACKR2-based approaches in the treatment and diagnosis of diseases might broaden our knowledge of pathogenesis to overcome complications.

## Author Contributions

AS: Conceptualization, Investigation, and Writing—Original Draft. FJ-N: Investigation and Writing—Original Draft. HM: Investigation and Writing—Original Draft. FE: Investigation and Writing—Review and Editing. MO: Investigation and Writing—Original Draft. AJ: Investigation and Writing—Original Draft. JN: Conceptualization, Writing—Original Draft, Methodology, and Supervision. All authors contributed to the article and approved the submitted version.

## Conflict of Interest

The authors declare that the research was conducted in the absence of any commercial or financial relationships that could be construed as a potential conflict of interest.

## Publisher’s Note

All claims expressed in this article are solely those of the authors and do not necessarily represent those of their affiliated organizations, or those of the publisher, the editors and the reviewers. Any product that may be evaluated in this article, or claim that may be made by its manufacturer, is not guaranteed or endorsed by the publisher.
